# Untangling the multiple timescales of Alzheimer's disease

**DOI:** 10.1002/alz70856_098560

**Published:** 2025-12-24

**Authors:** Lars Lau Raket

**Affiliations:** ^1^ Clinical Memory Research Unit, Lund University, Malmö, Sweden

## Abstract

**Background:**

Alzheimer's disease (AD) is a complex neurodegenerative disorder characterized by neuropathological changes leading to progressive cognitive decline. Understanding the multiple timescales of these changes is crucial for developing effective diagnostic and therapeutic strategies. This study aims to compare amyloid and tau PET clocks with novel staging methods that combine multiple biomarkers and clinical measures.

**Methods:**

The study utilized longitudinal data from 1064 Aβ‐positive subjects across the full AD continuum and 384 cognitively unimpaired Aβ‐negative participants, with a total follow‐up time of 6764 years. Amyloid and tau PET clocks were derived from available PET scans. Nonlinear mixed‐effects modeling was used to derive alternative stagings based on longitudinal clinical scales, amyloid PET, tau PET, and their combinations. Alignment quality was estimated using absolute Spearman correlation between estimated staging and unobserved clinical measures, as well as a range of fluid and imaging biomarkers. Deviations in predicted disease stage were compared across methods, and the uncertainty of each staging method across the AD continuum was assessed. The effect of age on staging quality was also investigated.

**Results:**

Staging based on nonlinear mixed‐effects modeling that combined amyloid PET, tau PET, and cognition was sensitive over a wider period of the AD continuum and produced better alignment of unobserved clinical measures and biomarkers compared to amyloid and tau PET clocks, which tended to overfit. The amyloid PET clock showed good alignment of measures in the preclinical and prodromal stages of the disease. The tau PET clock generally performed worse than amyloid PET, except for alignment of clinical scales in later disease stages. Adjusting for age improved alignment quality across all methods.

**Conclusion:**

Integrating multiple biomarker and clinical measures can improve AD staging. Amyloid and tau PET clocks are straightforward and user‐friendly tools for staging that have clear value, but they also have limitations in terms of their ability to consistently stage other disease‐related measures across the full AD continuum. Next‐generation amyloid and tau PET clocks could be derived based on more elaborate statistical models with more adequate regularization to avoid overfitting and the ability to include adjustments based on age and other readily available data.

